# Supplementary material for: Modelling Skylarks (Alauda arvensis) to Predict Impacts of Changes in Land Management and Policy: Development and Testing of an Agent-Based Model
Source: PLoS One. 2013 Jun 6;8(6):e65803. doi: 10.1371/journal.pone.0065803 (PMC3675089; doi:10.1371/journal.pone.0065803)
Supplement: Supporting Information S4 — The skylark ODdox as a zipped archive. (ZIP) [file pone.0065803.s004.zip › Skylark_ODdox/functions_0x62.html]

ALMaSS Skylark ODdox: Class Members


ALMaSS Skylark ODdox
 2.0


- Main Page
- Related Pages
- Classes
- Files

- Class List
- Class Index
- Class Hierarchy
- Class Members

- All
- Functions
- Variables
- Typedefs

- a
- b
- c
- d
- e
- f
- g
- h
- i
- j
- k
- l
- m
- n
- o
- p
- r
- s
- t
- u
- v
- w
- x
- y
- z
- ~

Here is a list of all class members with links to the classes they belong to:

### - b -

- BackTranslateEleTypes()
  : LE\_TypeClass
  , Landscape
- BackTranslateVegTypes()
  : Landscape
  , LE\_TypeClass
- BaitLocation()
  : BaitLocation
- BareRock()
  : BareRock
- BeetleBank()
  : BeetleBank
- BeetleBankAdd()
  : Landscape
- BeetleBankPossible()
  : Landscape
- BeforeStepActions
  : Population\_Manager
- BeginningOfMonth()
  : Population\_Manager
- BeginStep()
  : Skylark\_PreFledgeling
  , Skylark\_Female
  , Skylark\_Male
  , TALMaSSObject
  , TAnimal
  , Skylark\_Clutch
  , Skylark\_Nestling
- BinaryMapBase()
  : BinaryMapBase
- BitCount()
  : PositionMap
- BorderAdd()
  : Landscape
- BorderNeed()
  : Landscape
- BorderScan()
  : Landscape
- BorderScan2()
  : Landscape
- BorderStep()
  : Landscape
- BorderTest()
  : Landscape
- Bottom()
  : LowPriority< ELEMTYPE >
- BreedingPairsOutput()
  : Skylark\_Population\_Manager
  , Population\_Manager
- BreedingSuccessProbeOutput()
  : Skylark\_Population\_Manager
  , Population\_Manager
- BroodAge
  : Skylark\_Male
- BSuccess
  : Skylark\_Adult
- Building()
  : Building
- BuildingDesignationCalc()
  : Landscape
- BuiltUpWithParkland()
  : BuiltUpWithParkland
- BumpRunNum()
  : LE
- BurnStrawStubble()
  : Farm
- bx
  : Skylark\_struct
- by
  : Skylark\_struct


- Generated on Thu Jan 10 2013 13:15:39 for ALMaSS Skylark ODdox by
   1.8.1.1
